# Adriamycin, Vinblastine and Dacarbazine With Immunotherapy Achieves Complete Metabolic Response in a Patient With Classical Hodgkin Lymphoma and Dyskeratosis Congenita

**DOI:** 10.14740/jh2160

**Published:** 2026-03-10

**Authors:** Calum Slapnicar, Prateek Lala, Stephanie Lee, Sasan Zandi, Martina Trinkaus

**Affiliations:** a Temerty Faculty of Medicine, University of Toronto, Toronto, ON, Canada; b Department of Pharmacology and Toxicology, University of Toronto, Toronto, ON, Canada; c Division of Hematology/Oncology, St. Michael’s Hospital/Unity Health Toronto, Toronto, ON, Canada; d Laboratory Medicine and Pathobiology, University of Toronto, Toronto, ON, Canada; e Department of Molecular Genetics, University of Toronto, Toronto, ON, Canada

**Keywords:** Dyskeratosis congenita, Telomeropathy, Classical Hodgkin lymphoma, Brentuximab vedotin, BV-AVD, Chemotherapy toxicity, Radiation omission

## Abstract

Dyskeratosis congenita (DC) is a rare inherited telomeropathy characterized by defective telomere maintenance and an elevated risk of hematologic malignancies. Classical Hodgkin lymphoma (cHL) is a rare malignancy described in patients with DC, and optimal treatment remains undefined due to overlapping toxicities of standard ABVD (adriamycin, bleomycin, vinblastine, dacarbazine) and radiation therapy in this high-risk population. We present a 48-year-old man with longstanding thrombocytopenia who was diagnosed with DC based on clinical features and genetic testing. Two years post diagnosis, he developed stage IIA bulky cHL (nodular sclerosing, CD30+, Epstein-Barr virus (EBV)+). To mitigate pulmonary and myelotoxicity risks, he received a modified regimen of brentuximab vedotin (BV) combined with adriamycin, vinblastine, and dacarbazine (BV-AVD), with full omission of bleomycin. Treatment complications included peripheral neuropathy resulting in BV dose reduction and vinblastine discontinuation. Worsening thrombocytopenia led to discontinuation of dacarbazine. Interim imaging showed tumor regression, with post-treatment positron emission tomography with computed tomography (PET-CT) confirming complete metabolic response. Involved-site radiotherapy was omitted to minimize long-term risks of skin malignancy, local skin reactions and poor skin healing, in the context of DC. Post-treatment bone marrow evaluation showed no evidence of myeloid malignancy or lymphoma. This case demonstrates that modified BV-AVD can achieve complete metabolic remission in DC patients with cHL, while managing significant treatment-related toxicities. It underscores the critical need for individualized therapy in patients with DC and supports careful consideration of radiation omission to reduce secondary malignancy risk. These findings provide a potential therapeutic framework for managing Hodgkin lymphoma in patients with DC.

## Introduction

Dyskeratosis congenita (DC) is a rare telomere biology disorder (TBD), affecting approximately 1 in 1,000,000 individuals [1]. Patients with TBD have varied and progressive clinical manifestations due to aberrant telomere biology caused by the X-linked recessive, autosomal dominant, autosomal recessive, or *de novo* pathogenic germline variants in at least 18 different genes [2].

Clinically, DC classically presents with a mucocutaneous triad comprising nail dystrophy, oral leukoplakia, and reticular hyperpigmentation—features that are present in approximately 80% of cases [3]. Non-cutaneous manifestations include progressive bone marrow failure (up to 90% of individuals), pulmonary and hepatic fibrosis, as well as gastrointestinal and genitourinary tract strictures [4]. A key diagnostic hallmark of DC is the presence of age-adjusted shortened telomeres in blood leukocytes [4]. This telomere attrition contributes to genomic instability and hematopoietic stem cell exhaustion, which underlie the markedly increased risk of hematologic malignancies, particularly myelodysplastic syndrome (MDS; 2,500-fold risk) and acute myeloid leukemia (AML; 196-fold risk) [1, 3, 5]. Although rare, classical Hodgkin lymphoma (cHL) has been reported in DC, with three reported cases to date [6–8]. One of these cases represented a man who achieved remission with an unspecified regimen of combined radiation–chemotherapy, ultimately dying years later from a gastric adenocarcinoma [6]; the other cases did not include details of the presenting illness, treatment or outcome. One cohort reported a prevalence of cHL among DC patients at 0.68% (1 in 148) [8], compared to the general population prevalence of 0.07% [9], albeit biased by lack of availability to larger DC cohorts for more accurate prevalence rates.

The current first-line treatment for limited-stage cHL is a combination chemotherapy regimen known as ABVD—comprising adriamycin, bleomycin, vinblastine, and dacarbazine—administered for two to six cycles based on disease stage and interim positron emission tomography (PET) scan response. This is often followed by involved-site radiation therapy (ISRT), pending on disease bulk. In the general population, ABVD therapy alone achieves 5-year progression-free survival (PFS) and overall survival (OS) rates of approximately 87% and 94%, respectively [10]. However, several components of ABVD pose increased risk in DC: bleomycin is linked to severe pulmonary fibrosis [11]; dacarbazine can worsen myelotoxicity and genotoxicity [12]; and ISRT carries added risk for radiation-induced skin sarcomas and other secondary neoplasms and organ toxicities in patients with early-stage Hodgkin lymphoma (ESHL) [13, 14].

Emerging data from several recent trials have highlighted the efficacy of monoclonal antibody-based therapies in cHL, particularly in advanced-stage disease [15, 16]. One such agent, brentuximab vedotin, an anti-CD30 antibody-drug conjugate, when combined with AVD (adriamycin, vinblastine, dacarbazine), has demonstrated superior PFS, OS and less pulmonary toxicity compared to traditional ABVD in stage III/IV cHL, sustained over 7 years [15, 16]. This bleomycin-sparing regimen (BV-AVD) may offer a more favorable therapeutic profile for patients with underlying telomeropathies.

This report describes a case of a patient with DC presenting with stage IIA bulky cHL, who achieved a successful response to BV-AVD treatment, representing the first such case reported in a DC patient.

## Case Report

A 48-year-old man with a previous medical history including type 2 diabetes, hypertension and dyslipidemia presented to the hematology clinic with 3 years of asymptomatic thrombocytopenia. Family history was notable for a first cousin’s daughter who died of childhood leukemia. Relevant findings on physical exam included non-tender left infraclavicular fullness. Initial blood work demonstrated hemoglobin 132 g/L (mean corpuscular volume (MCV) 99), white blood cell count 5.8 × 10^9^/L, and platelet count 77 × 10^9^/L with normal biochemistries and viral workup. A provisional diagnosis of immune thrombocytopenia was assigned.

The patient underwent magnetic resonance imaging (MRI) to investigate the infraclavicular fullness which demonstrated pronounced left axillary and retropectoral lymphadenopathy, with the largest node measuring approximately 55 × 29 mm. Axillary node resection revealed reactive, nonspecific features and was negative for lymphoma.

**Figure 1:**
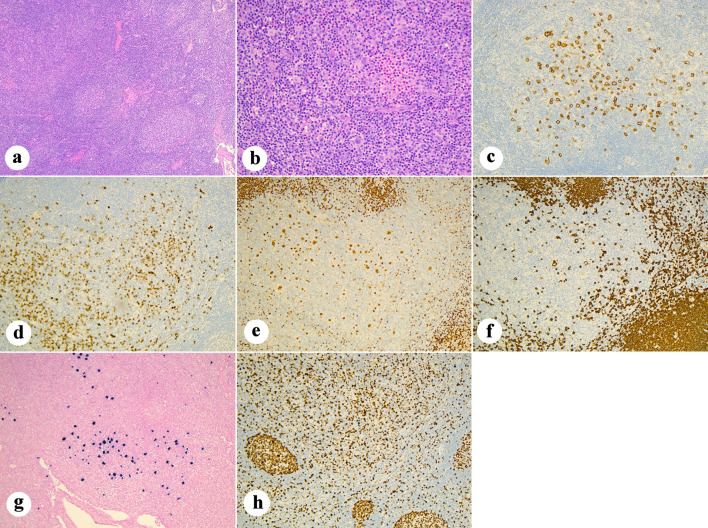
Histopathologic and immunophenotypic features of classical Hodgkin lymphoma in dyskeratosis congenita: (a) Low-power view of lymph node architecture (H&E, × 5) showing partial effacement by nodular proliferation. (b) Higher-power field (H&E, × 20) demonstrating scattered large, atypical Hodgkin/Reed–Sternberg cells with prominent nucleoli in an inflammatory background rich in small lymphocytes and eosinophils. (c) CD30 shows strong membranous and paranuclear positivity in the large, atypical cells (× 10). (d) CD15 highlights the same large, atypical cells with cytoplasmic/membranous staining (× 10). (e) PAX5 demonstrates weak nuclear staining in the neoplastic cells with strong positivity in background B cells (× 10). (f) CD20 shows loss of expression in the Hodgkin cells (× 10). (g) EBER *in situ* hybridization reveals nuclear positivity within Hodgkin cells (× 10). (h) Ki-67 immunostaining shows a high proliferative index within the atypical cells and peripheral reactive germinal centers (× 10). H&E: hematoxylin and eosin; PAX5: paired box 5; EBER: Epstein-Barr virus-encoded small RNAs.

**Figure 2:**
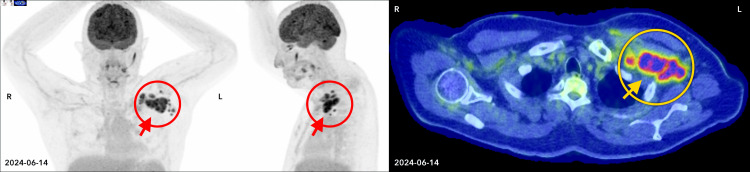
Pre-BV-AVD imaging. Pre-BV-AVD FDG positron emission tomography-computed tomography (PET-CT) maximum intensity projection (axial and coronal, left) and transverse section (right) demonstrating an intensely FDG-avid left retropectoral lymph node conglomerate (indicated by arrows). BV-AVD: brentuximab vedotin, adriamycin, vinblastine, and dacarbazine; FDG: fluorodeoxyglucose.

Further evaluation of his clinical picture revealed the presence of reticular hyperpigmentation, nail dystrophy, hepatic fibrosis, and ureteral stenosis. A bone marrow examination was performed, showing a normal male karyotype and no abnormalities on fluorescence *in situ* hybridization. Morphology showed occasional and mild erythroid and megakaryocytic atypia, not meeting diagnostic threshold for MDS. Cellularity measured 10–40%. No sign of Hodgkin lymphoma was present. A myeloid next-generation sequencing (NGS) panel showed a *TERT* c.1891C>T p.(Arg631Trp) at a variant allele frequency (VAF) of 51%, which was signed out as Tier 3; NGS also showed a *TERT* c.–57 A>C 15% (Tier 1) and a *TET2* c.167C>G p.(Ser56Cys) 49% (Tier 3). Telomere length assay confirmed age-adjusted telomere length < 1% in both lymphocytes and granulocytes. He underwent testing on cultured skin fibroblasts for 187 genes associated with inherited bone marrow failure syndromes, which detected the same *TERT* c.1891C>T variant identified in the bone marrow. The constellation of clinical symptoms, telomere length and genetic workup was in keeping with DC, and the *TERT* c.1891C>T variant was thought to be likely pathogenic [17]. A larger section of the left retropectoral mass was excised, and histopathologic examination demonstrated partial architectural effacement with scattered Hodgkin/Reed–Sternberg cells in a mixed inflammatory background. The atypical cells were CD30- and CD15-positive, Epstein-Barr virus-encoded small RNAs (EBER)-positive, showed weak paired box 5 (PAX5) expression with loss of CD20, and a high Ki-67 proliferative index, consistent with cHL, nodular sclerosis subtype (Fig. 1). The combination of CT, MRI and fluorodeoxyglucose (FDG)-PET; Fig. 2) studies yielded a diagnosis of stage IIA bulky disease (8.2 × 6.7 × 6.2 cm).

Amongst multidisciplinary subspecialists, a consensus decision was reached to initiate a regimen of modified BV-AVD to balance efficacy with toxicity. Nivolumab, a programmed cell death 1 (PD-1) checkpoint inhibitor studied in advanced HL was not available for possible provision. BV (1.2 mg/kg), adriamycin (25 mg/m^2^), dose-reduced vinblastine (3 mg/m^2^), and dacarbazine (188 mg/m^2^) were administered for a total of four cycles. Specifically, bleomycin was omitted to mitigate the risk of pulmonary fibrosis; dacarbazine and vinblastine were dose-reduced by 50% due to concerns of cumulative myelotoxicity and overlapping risk of neuropathy with BV, respectively. Despite early dose modifications, the patient experienced ongoing toxicities over the course of BV-AVD, prompting further treatment adjustments. Dacarbazine was discontinued after cycle 1, day 15 due to worsening thrombocytopenia (*nadir* 22 × 10^9^/L), and vinblastine was stopped after cycle 2, day 1 due to grade 2 peripheral neuropathy (as defined by reduced sensation to his hands and reduced motor function affecting basic instrumental activities of daily living) [18]. Anemia progressively worsened during BV-AVD treatment, declining from a baseline of 118 g/L to a *nadir* of 84 g/L, in the setting of persistent hematuria subsequently attributed to urethral strictures. This was managed supportively with subcutaneous epoetin alfa. To minimize radiation exposure, interval MRI was performed after cycle 2, which demonstrated a reduction in axillary lymph node size, with one representative node decreasing from 2.9 cm to 1.7 cm in short axis.

**Figure 3:**
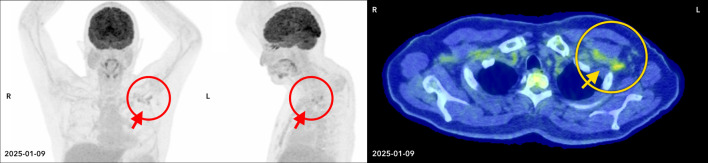
Post-BV-AVD imaging. Post-BV-AVD FDG positron emission tomography-computed tomography (PET-CT) maximum intensity projection (axial and coronal, left) and transverse section (right) showing interval reduction in nodal size and FDG uptake (indicated by arrows). BV-AVD: brentuximab vedotin, adriamycin, vinblastine, and dacarbazine; FDG: fluorodeoxyglucose.

Post-cycle 4 PET with computed tomography (PET-CT) demonstrated a significant interval decrease in the size and metabolic activity of previously involved above-diaphragmatic lymph nodes, with residual left axillary nodes showing uptake at the level of the hepatic background (Deauville 3 [19]), consistent with a complete metabolic response to treatment (Fig. 3). The role of ISRT was discussed with the patient and at tumor boards; ultimately, it was omitted due to concerns about heightened toxicity in the context of his DC. As a result of this patient’s pancytopenia observed throughout BV-AVD, post-treatment bone marrow examination was performed. The aspirate demonstrated no evidence of clonal lymphoid proliferation, with no monotypic B cells or abnormal T cells detected on flow cytometry. CD34^+^ blasts comprised less than 1% of cells. The trephine showed a mildly hypocellular marrow with approximately 40% cellularity, and preserved trilineage hematopoiesis. Cytogenetic analysis revealed a normal karyotype, and NGS showed the same *TERT* mutations at the same VAF, with no new mutations compared to prior testing.

The patient continues with frequent clinical and bone marrow surveillance. Peripheral neuropathy persisted for several months post-BV-AVD, with eventual resolution; normalization of anemia and persistent thrombocytopenia (36–51 × 10^9^/L) remains a notable concern, with repeat bone marrow evaluations remaining negative for malignancy, and positive for mild megakaryocytic hypoplasia.

## Discussion

We present the case of a now 51-year-old man newly diagnosed with DC, who was subsequently diagnosed with stage IIA bulky cHL, and successfully treated with four cycles of modified BV-AVD who now remains in complete remission for 12 months.

Historically, cHL has only rarely been associated with inherited telomeropathies, and there is no published consensus on HL-directed treatment in patients with DC. In this patient, bone marrow NGS revealed a pathogenic somatic *TERT* promoter mutation (*TERT* c.–57 A>C 15%). While this specific oncogenic variant has not been directly linked to cHL, such acquired mutations may reflect clonal expansion of a hematopoietic progenitor under telomere stress, with potential contribution to lymphoid lineages and malignant transformation [20].

The patient was initiated on a modified BV-AVD regimen, omitting bleomycin to avoid pulmonary toxicity. Common BV-AVD toxicities include neutropenia (44%) and peripheral neuropathy (54%) [21]. While peripheral neuropathy has been reported in DC, it is not a hallmark feature [22] and is a known adverse effect of BV and vinblastine. Standard practice to avoid neuropathy requires BV dose adjustment alone [16, 21, 23], but in patients with elevated neurotoxicity risk, vinblastine omission may be reasonable, as supported by regimens like BV-N-AD (BV, nivolumab, adriamycin, dacarbazine), which avoid dual microtubule inhibition while preserving efficacy [24]. Nivolumab, a PD-1 checkpoint inhibitor, was not available for provision during this patient’s treatment course and hence not considered for inclusion. Neutropenia was managed with granulocyte colony-stimulating factor (G-CSF) support, maintaining absolute neutrophil counts above 0.5 × 10^9^/L throughout therapy. Both neuropathy and neutropenia improved post-treatment, consistent with previously reported BV-AVD cohorts [16, 21]. Anemia and thrombocytopenia—known AEs of dacarbazine—were also observed. Dacarbazine was dose-reduced by 50% at initiation, then discontinued after cycle 1, day 15 due to worsening hematuria and thrombocytopenia. While anemia is common with BV-AVD and ABVD (21% and 10%), thrombocytopenia is not typically reported [16]. In this case, its persistence despite dacarbazine cessation is multi-factorial from both his chemotherapy and underlying DC.

In our case, ISRT was omitted following a complete metabolic response after four cycles of BV-AVD. This decision was guided by the risk of excessive toxicity from radiation therapy, despite literature showing conflicting PFS with radiotherapy omission in ESHL. The RAPID trial included patients with stage IA and IIA disease, including some with unfavorable risk features, though it did not stratify outcomes by risk and excluded those with bulky disease [25]. Among patients with negative PET scans after three cycles of ABVD, omission of ISRT resulted in a slightly lower 3-year PFS (90.8% vs. 94.6%), and noninferiority was not demonstrated [25]. In contrast, Kumar et al studied patients with early-stage, unfavorable-risk cHL—including bulky disease—and observed high PET-4 negativity rates and a 2-year PFS of 96.6% in those treated with BV-AVD without radiotherapy [21]. These outcomes were comparable to cohorts that did receive radiotherapy, reinforcing the potential safety of omitting radiation in select patients [21]. One limitation in applying the findings of Kumar et al to our case is that only 1.3% of total doses in their cohort were dose-reduced, primarily BV, whereas our patient required dose reductions of BV, and discontinuation of vinblastine and dacarbazine due to toxicity. Taken together, these findings support an individualized approach to radiotherapy in ESHL, particularly in patients with increased long-term treatment toxicity risk, such as those with DC.

Subsequent genetic testing of the family identified an asymptomatic sister who carried the same heterozygous *TERT* variant, c.1891C>T p.(Arg631Trp) confirmed on skin fibroblasts; parental testing is currently pending.

This case supports the feasibility of bleomycin-sparing, individualized BV-AVD in DC-associated cHL and highlights a rational basis for radiation omission after PET complete response (CR) in inherited bone-marrow failure syndromes.

## Data Availability

The data supporting the findings of this study are available from the corresponding author upon reasonable request.

## Abbreviations

DC: dyskeratosis congenita
cHL: classical Hodgkin lymphoma
ABVD: adriamycin, bleomycin, vinblastine, and dacarbazine
ISRT: involved-site radiation therapy
PFS: progression-free survival rate
OS: overall survival
ESHL: early-stage Hodgkin lymphoma
BV: brentuximab vedotin
AVD: adriamycin, vinblastine, dacarbazine
BV-AVD: brentuximab vedotin, adriamycin, vinblastine, and dacarbazine
PET-CT: positron emission tomography with computer tomography
TBD: telomere biology disorder
MDS: myelodysplastic syndrome
NGS: next-generation sequencing
G-CSF: granulocyte colony-stimulating factor
AE: adverse event
